# Liquid‐Assisted Single‐Layer Janus Membrane for Efficient Unidirectional Liquid Penetration

**DOI:** 10.1002/advs.202103765

**Published:** 2021-11-10

**Authors:** Zhihong Zhao, Yuzhen Ning, Shuang Ben, Xudong Zhang, Qiang Li, Cunming Yu, Xu Jin, Kesong Liu, Lei Jiang

**Affiliations:** ^1^ Key Laboratory of Bio‐Inspired Smart Interfacial Science and Technology School of Chemistry Beihang University Beijing 100191 P. R. China; ^2^ School of Mechanical Engineering and Automation Beihang University Beijing 100191 P. R. China; ^3^ Research Institute of Petroleum Exploration and Development PetroChina Beijing 100083 China; ^4^ Beijing Advanced Innovation Centre for Biomedical Engineering Beihang University Beijing 100191 China

**Keywords:** auxiliary liquids, backflow prevention, high pressure differential, single‐layer Janus membranes, unidirectional liquid penetration

## Abstract

Unidirectional liquid penetration plays an important role in many fields, such as microfluidic devices, biological medical, liquid printing, and oil/water separation. Although there are some progresses in the liquid unidirectional penetration using a variety of Janus membranes with anisotropic wettability, it still remains a great difficulty for single‐layer Janus membranes with straight pore to balance spontaneous liquid penetration in positive direction and superior liquid resistance in the reverse direction. Herein, a liquid‐assisted strategy for single‐layer Janus membrane is developed, which can efficiently decrease the critical breakthrough pressure from superhydrophobic side to hydrophilic side and show little influence on that in the reverse direction. Consequently, unidirectional water penetration with high hydraulic pressure difference can be achieved. The Laplace pressure change along the thickness of the single‐layer Janus membranes is further discussed, and the mechanism by which the auxiliary liquid decreases the critical breakthrough pressure is revealed. Furthermore, this Janus membrane with unidirectional water penetration “diode” performance can be used to prevent liquid backflow in intravenous transfusion. It is believed that this work can open an avenue for people to design single‐layer Janus membrane with high pressure difference and find wide applications in unidirectional liquid transport.

## Graphical abstract

A liquid‐assisted single‐layer Janus membrane is developed, which can efficiently decrease the critical breakthrough pressure from superhydrophobic side to hydrophilic side and show little influence on that in the reverse direction, thereby achieving unidirectional water penetration with high pressure difference. This liquid diode Janus membrane with high pressure difference can solve the problem of blood backflow during intravenous transfusion.

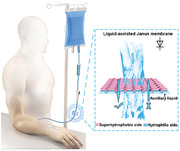

## 1 Introduction

Janus membranes with opposite wettability on each side attract widespread interest over the past years due to their unique unidirectional penetration property, i.e., liquid can penetrate effortlessly in the positive direction (hydrophobic side to hydrophilic side), whereas the reverse transport (hydrophilic side to hydrophobic side) is blocked.^[^ ^1^ ^]^ Such directional liquid transport without an increased energy penalty shows great potential in many fields, such as functional textiles,^[^ ^1d^ ^]^ fog collection,^[^ ^2^ ^]^ oil/water separation,^[^ ^3^ ^]^ sensor,^[^ ^4^ ^]^ etc. Currently, the Janus membranes can be categorized into two main types: multilayer Janus membranes containing two closely connected layers with opposite wettability and single‐layer Janus membrane with a wettability gradient along the material thickness.^[^ ^5^ ^]^ Multilayer Janus membranes are obtained by manufacturing each side of the membrane separately and then combining them together. Although the preparation method is simple, the interfacial bonding is relatively weak due to the incompatibility between the two layers.^[^ ^6^ ^]^ Single‐layer Janus membranes are favored due to the structural integrity and unicity. So far a large number of methods have been developed to fabricate the single‐layer Janus membrane, including single‐faced photodegradation,^[^ ^7^ ^]^ single‐faced photo‐crosslinking,^[^ ^3^ ^]^ floated deposition,^[^ ^8^ ^]^ vapor treatment,^[^ ^1a^ ^]^ etc. However, limited by the membrane structure, the positive critical breakthrough pressure and reverse critical breakthrough pressure of single‐layer Janus membrane with straight pore are mutually restricted. That is to say, the spontaneous liquid transport in the positive direction usually means weak penetration resistance in the reverse direction.^[^ ^9^ ^]^ These single‐layer Janus membranes with low hydraulic pressure difference are more like a quasi‐unidirectional penetration, which limit their applications. Therefore, it is urgently needed to develop a strategy for the single‐layer Janus membrane to balance spontaneous liquid transport capacity in positive direction and superior liquid blocking performance in reverse direction.

Nature uses solid‐liquid composite materials instead of pure solid materials to construct many unique functions, such as antibacterial and antifouling of eyes, lubricity and pressure resistance of lungs, the shape‐adapting soles of insect feet, and slippery surface of Nepenthes.^[^ ^10^ ^]^ Using the flexibility, diffusion, molecular smoothness, and self‐adaptability of liquids, solid‐liquid composite structure offers a unique combination of dynamic and interfacial behavior.^[^ ^11^ ^]^ This liquid‐assisted strategy can break some limitations derived from intrinsic characteristics of solid materials. Herein, inspired by the solid‐liquid composite materials in nature, we construct a liquid‐assisted single‐layer Janus membrane by simply introducing a liquid layer on the hydrophilic side of the conventional Janus membrane. A series of Janus membranes with different hydrophilization depth were prepared by adjusting single‐face alkali treatment time. By comparing the liquid penetration performance of liquid‐assisted Janus membrane and conventional single‐layer Janus membrane, we theoretically and experimentally demonstrate that the auxiliary liquid can increase the pressure difference between the two sides of the Janus membrane. The liquid diode Janus membrane with high pressure difference can be used to prevent backflow and is expected to solve the problem of blood backflow during intravenous transfusion. This work will further strength our understanding of designing single‐layer Janus membrane with high pressure difference and find wide applications in various fields, e.g., biomedical engineering, fluid transportation, and energy engineering.

## 2 Results and Discussion

### 2.1 Preparation of Liquid‐Assisted Janus Membrane

**Figure 1:**
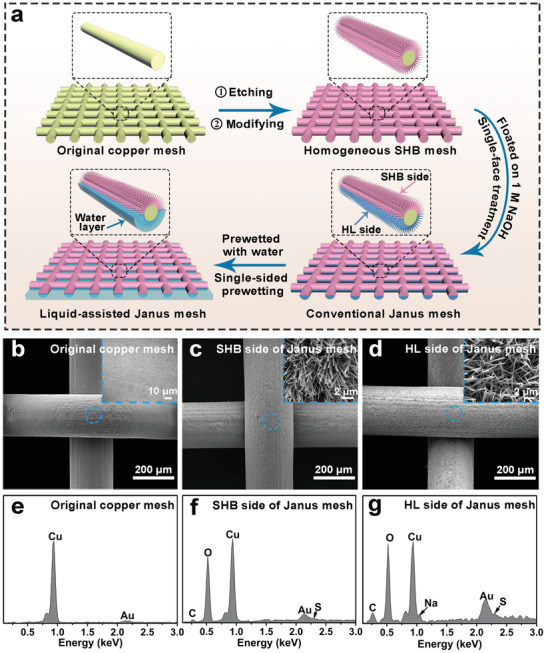
Preparation and characterization of liquid‐assisted Janus membrane. a) Schematic illustration of the preparation process of liquid‐assisted Janus membrane. The conventional Janus membrane is prepared by following the processes of ammonia alkali etching, superhydrophobic modification with thiol (SH(CH_2_)_9_CH_3_ and SH(CH_2_)_10_COOH) and single‐face hydrophilization treatment with NaOH solution. The liquid‐assisted Janus membrane is obtained by prewetting the hydrophilic side of the conventional Janus membrane with water. b–d) SEM characterization. b) The original copper mesh possesses a relatively smooth surface. For the Janus membrane obtained after asymmetric hydrophilization treatment (≈10 min), both the c) superhydrophobic (SHB) side and d) hydrophilic (HL) side maintained micro/nano multiscale surface structures. The insets are the magnified SEM image. e–g) Surface elements analysis. EDX spectra of e) original copper mesh, f) SHB side, and g) HL side of Janus membrane. The peaks representing C and S elements appeared on copper mesh modified with thiol, and the peak of Na element was observed on the HL side of Janus membrane.

The fabrication process of liquid‐assisted Janus membrane is shown in **Figure** **1**a. The homogeneous superhydrophobic (SHB) mesh is fabricated by chemically etching and hydrophobization modification with thiol (SH(CH_2_)_9_CH_3_ and SH(CH_2_)_10_COOH). Then, the obtained SHB mesh was placed on the surface of NaOH solution. The side of SHB mesh contacting with NaOH solution turned to be hydrophilic, while the other side without contacting with NaOH solution maintained its superhydrophicity. After that, the hydrophilic (HL) side was prewetted by water and a liquid‐assisted Janus membrane was fabricated. The surface morphologies of original copper surface, SHB side surface, and HL side surface can be observed by Figure 1b–d. Compared with the smooth surface structure of original copper surface (Figure 1b), the SHB one was composed of needle‐like micro/nanostructures (Figure 1c). After asymmetric hydrophilization with NaOH solution, the morphology of micro/nanostructures on HL surface was little changed (Figure 1d). Energy‐dispersive X‐ray spectroscopy (EDX) was also used to analysis the element composition of these surfaces. As shown in Figure 1e, only Cu peak can be observed on original copper surface. The Au element comes from the gold spraying before sample testing. The appearance of C and S peaks on both sides of Janus membrane indicates that thiol has been successfully modified on the membrane surface. Furthermore, compared with the surface composition of the SHB side (Figure 1f), the peak representing Na element appears on HL side (Figure 1g), which means that the floated alkali treatment only changes the contact side surface composition while the other side remains intact. In summary, different chemical compositions lead to the opposite wettability of Janus membrane on both sides.

**Figure 2:**
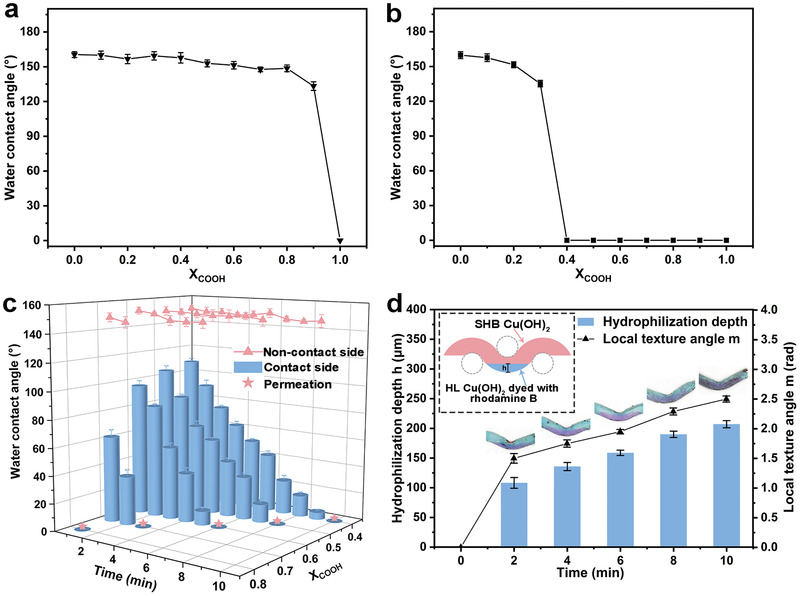
Surface wettability. a) Water contact angle on surface modified with different *X*_COOH_ (mole fraction of HS(CH_2_)_10_COOH in the mixed thiol solution). b) Water contact angle as a function of *X*_COOH_ on the surface after 1 m NaOH treatment for 10 min. Alkali treatment can change the surface (*X*_COOH_ = 0.4–0.9) wettability from superhydrophobicity to hydrophilicity. c) The relationship between the wettability of both sides of the Janus membrane modified with different *X*_COOH_ and the alkali treatment time. The contact side represents the one side of membrane contacting with NaOH solution, and the noncontact side represents the other side of the membrane without contacting with NaOH solution. Alkali treatment only makes the one side in contact with the NaOH solution hydrophilic, while the other side of the membrane remains superhydrophobic. Permeation represents that the droplets dropped on the noncontact side would spontaneously penetrate through the Janus membrane during the contact angle test. d) The relationship between hydrophilization depth *h*, local texture angle *m*, and alkali treatment time. With the increase of alkali treatment time, hydrophilization depth *h* and local texture angle *m* gradually increase. The insets are the copper wire surface with different hydrophilization modification time, respectively. The HL side of the copper wire surface is dyed with rhodamine B.

Using the hydrophobicity of SH(CH_2_)_9_CH_3_ (alkyl) and the tunable wettability of SH(CH_2_)_10_COOH (carboxyl),^[^ ^12^ ^]^ we firstly fabricated homogeneous SHB membrane, and then one side of membrane was selectively deprotonated into hydrophilicity through alkali treatment to finally obtain Janus membrane. To determine the alkyl/carboxyl ratio, the relationship between surface wettability and various alkyl/carboxyl ratios is discussed. As shown in **Figure** **2**a, when the mole fraction of carboxyl (*X*_COOH_) is less than 1, the surface performs superhydrophobicity. Alkali treatment can change the surface wettability due to the presence of carboxyl. When the alkali treatment time is 10 min, the surfaces modified with *X*_COOH_ = 0.4–0.9 change from superhydrophobicity to superhydrophilicity, while the surfaces modified with *X*_COOH_ = 0–0.3 maintain hydrophobic property (Figure 2b). In addition, further extension of the alkali treatment time has little effect on the wettability of these surfaces (*X*_COOH_ = 0–0.3). Considering only the change in wettability, alkyl/carboxyl ratios between *X*_COOH_ = 0.4–0.9 seem to be used to prepare Janus membranes with opposite wettability on each side. To determine the optimal *X*_COOH_, we further explore the wettability on both sides of the membrane obtained under different alkali treatment time (Figure 2c). The contact angle on the surface modified with *X*_COOH_ = 0.9 is not shown in Figure 2c, because the surface becomes superhydrophilic upon contact with the alkali solution. With the decrease of *X*_COOH_, the alkali treatment time required for the preparation of the Janus membrane with unidirectional permeability gradually increases. Long alkali treatment time is beneficial for us to control the hydrophilization depth. Therefore, the optimal *X*_COOH_ is set to be 0.4 for constructing the Janus membrane with unidirectional permeability. For the membrane prepared at the optimal ratio (*X*_COOH_ = 0.4), we quantified the relationship between the alkali treatment time and the hydrophilization depth *h*. Here rhodamine B is used to mark the hydrophilic part. As shown in the insets in Figure 2d, the thickest position of the hydrophilic part on the single copper wire represents the hydrophilization depth *h*. The local texture angle *m* corresponding to the hydrophilization depth *h* can be obtained according to Equation (1) (Figure S1, Supporting Information)

(1) $$m=\mathrm{arc}\cos\left(\frac{R-h}{R}\right)$$

*R* is the wire radius. The corresponding relation between hydrophilization modification time, hydrophilization depth *h*, and local texture angle *m* is shown in Figure 2d.

### 2.2 Water Unidirectional Penetration

**Figure 3:**
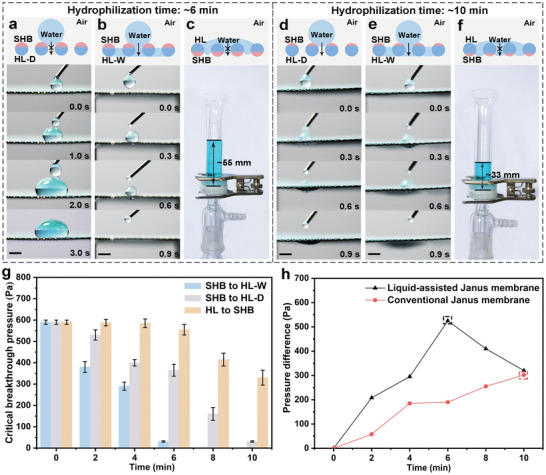
Water unidirectional penetration. Through the control of single‐face hydrophilization treatment time, Janus membrane with different hydrophilization depth were obtained. a) When the single‐face hydrophilization treatment time is ≈6 min, water cannot penetrate the conventional Janus membrane from the SHB to HL‐D (no prewetting treatment on the HL side) direction and b) can spontaneously penetrate liquid‐assisted Janus membrane from SHB to HL‐W (prewetting treatment on the HL side) direction. c) Water is blocked from HL to SHB direction, and the height of the water column can reach about 55 mm. d,e) When the single‐sided hydrophilization treatment time is ≈10 min, water can spontaneously penetrate the Janus membrane from SHB to HL direction, regardless of whether the HL side is prewetted by water. f) Water is intercepted in the direction of HL to SHB, and the height of the water column is reduced to ≈33 mm. The water is dyed by methylene blue. Scale bars: 2.5 mm. g) The effect of hydrophilization modification time on critical breakthrough pressure of the liquid‐assisted Janus membrane and conventional Janus membrane. SHB to HL‐W represent the positive direction of the liquid‐assisted Janus membrane, and SHB to HL‐D represent the positive direction of the conventional Janus membrane. HL to SHB represent the reverse direction of the conventional Janus membrane and liquid‐assisted Janus membrane. h) The relationship between hydrophilization modification time and the pressure difference. The marked pressures are the pressure difference of liquid‐assisted Janus membrane with unidirectional penetration performance and conventional Janus membrane with unidirectional penetration performance, respectively. Here copper mesh with pore diameter of ≈460 µm is used for testing.

The position of the hydrophilic–hydrophobic interface within the membrane greatly affects the critical breakthrough pressure.^[^ ^13^ ^]^ Here, the position of the hydrophilic–hydrophobic interface was adjusted by tuning the single‐sided alkali treatment time. When the single‐sided hydrophilization time is ≈6 min, for conventional Janus membrane (no prewetting treatment on the HL side, HL‐D), water droplets cannot penetrate through the membrane in the direction of SHB to HL‐D (**Figure** **3**a; Movie S1, Supporting Information), while for the liquid‐assisted Janus membrane (prewetted by water on the HL side, HL‐W), single water droplet would spontaneously penetrate to the HL‐W side as soon as it contacts the SHB side (Figure 3b; Movie S2, Supporting Information). Here, the pressure generated by single water droplet is about 30 Pa. In the reverse HL to SHB direction, water was blocked with critical water column height up to 55.5 ± 2.5 mm (Figure 3c). The auxiliary liquid does not affect the reverse critical breakthrough pressure. When the single‐sided hydrophilization time was increased to ≈10 min, single water droplet can spontaneously penetrate the Janus membrane from SHB to HL direction, regardless of whether the HL side is prewetted by water (Figure 3d,e; Movies S3 and S4, Supporting Information). But the critical water column height was reduced to 33.5 ± 4.0 mm in reverse direction (Figure 3f). Figure 3g further displays the relationship between alkali treatment time and critical breakthrough pressure of the Janus membrane. As the single‐sided hydrophilization treatment time increases, the critical breakthrough pressure from SHB to HL gradually decreases, while the critical breakthrough pressure from HL side to SHB side firstly remains unchanged and then gradually decreases. In this process, the critical breakthrough pressure from SHB to HL‐W (liquid‐assisted Janus membrane) is always less than that from SHB to HL‐D (conventional Janus membrane). The pressure difference in positive and reverse direction of the liquid‐assisted Janus membranes and conventional Janus membrane under different hydrophilization modification time is further compared (Figure 3h). Due to the reduction effect of the auxiliary liquid on the positive breakthrough pressure, the pressure difference of the liquid‐assisted Janus membrane is higher than that of the conventional Janus membrane. Furthermore, the unidirectional penetration always took place at the point where the pressure difference reached the maximum. Compared with the conventional Janus membrane, the liquid‐assisted Janus membrane only needs a shorter hydrophilization modification time to realize the unidirectional water penetration. Thus the liquid‐assisted Janus membranes possess high pressure difference in unidirectional water penetration. The unidirectional permeability performance of Janus membranes with different pore sizes has been further studied (Figure S2, Supporting Information). Compared with conventional Janus membranes with different pore sizes, the liquid‐assisted Janus membranes show water unidirectional penetration performance with higher pressure difference.

### 2.3 Unidirectional Liquid Transport Mechanism

The unidirectional liquid penetration is caused by the difference in critical breakthrough pressure when liquid penetrates from two different sides. For the single‐layer Janus membrane with straight pore, the Laplace pressure Δ*P* on the arch‐shaped liquid surface follows Equation (2)

(2) $$\Delta P=-\frac{2\gamma }{r}=\frac{2\gamma \sin\left(\theta \left(\phi \right)-\phi \right)}{R\left(\sin\phi -D^{*}\right)}\;$$

where *r* is curvature radius of the curved liquid surface, *γ* is interfacial tension, *θ*(*ϕ*) represents the contact angle, *ϕ* is the local texture angle (*ϕ*  =  *θ*(*ϕ*) − *δθ*, *δθ* is the sagging angle of liquid), D* = (*R* + *D*)/*R* is the geometrical spacing ratio, *R* is the wire radius, and 2*D* is the distance between the two adjacent wires. When the liquid penetrates from two different directions, the expression of *θ*(*ϕ*) is different. Here, the relationship between the positive contact angle (*θ*_P_(*ϕ*)) and the reverse contact angle (*θ*_r_(*ϕ*)) follows Equation (3)^[^ ^14^ ^]^

(3) $$\theta_{P}\;\left(\phi \right)=\theta_{r}\;\left(\pi -\phi \right)$$

**Figure 4:**
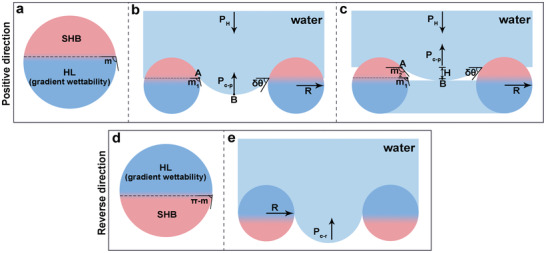
Mechanism of water unidirectional penetration on Janus membranes. The wettability changes along the cylinder surface in the a) positive and d) reverse direction. In the positive direction, the cylinder surface is divided into two areas: superhydrophobic (SHB) area within *ϕ* ∈ [*π*, *m*) and HL (gradient wettability) area within *ϕ* ∈ [*m*, 0]. In the reverse direction, the wettability along cylinder surface is successively HL (gradient wettability) area within *ϕ* ∈ [*π*, *π* − *m*] and SHB area within *ϕ* ∈ (*π* − *m*, 0]. Here, *ϕ* is the local texture angle. b) Water unidirectional penetration on a conventional Janus membrane. For conventional Janus membrane with HL (gradient wettability) within *ϕ* ∈ [*m*_1_,0], the critical breakthrough pressure in the positive direction (*P*_c − p_) is reached when points A (solid‐liquid‐gas three‐phase contact point) reaches the position where *ϕ* is equal to *m*_1_. Once the hydraulic pressure (*P*_H_) exceeds *P*_c − p_, the positive liquid penetration can be achieved. c) Water unidirectional penetration on a liquid‐assisted Janus membrane with gradient wettability within *ϕ* ∈ [*m*_1_,0]. Compared to the conventional Janus membrane, the *P*_c − p_ of liquid‐assisted Janus membrane is reduced through the contact of point B (lowest point of liquid meniscus) with the prewetted water layer. e) *P*_c − r_ is the critical breakthrough pressure in the reverse direction. The *P*_c − r_ of liquid‐assisted Janus membrane remains consistent with that of the conventional Janus membrane.

As the Janus membrane is prepared by single‐face hydrophilization modification of homogeneous SHB membrane, the wettability of cylinder surface can be divided into two parts along the positive direction (**Figure** **4**a): SHB area within *ϕ* ∈ [*π*, *m*) and HL (gradient wettability) area within *ϕ* ∈ [*m*, 0]. The surface wettability along the reverse direction is shown in Figure 4d. Here, *m* varies within[0, *π*]. The *m* is related to the hydrophilization modification time, and a long hydrophilization modification time means a large *m* value (Figure 2d). To simplify the discussion, we assume that there is a linear relationship between *ϕ* and *θ*(*ϕ*).^[^ ^14^ ^]^ The contact angle on the SHB side is $\frac{5\pi }{6}$. Therefore, the expression of Δ*P* in the positive and reverse directions can be obtained, i.e., Δ*P*_P_ and Δ*P*_r_

(4) $$\Delta P_{P}=\left\{\begin{array}{cc} \frac{2\gamma \sin\left(\frac{5\pi }{6}-\phi \right)}{R\left(\sin\phi -D^{*}\right)} & \phi \in \left[\pi \right.,\left.m\right)\\ \frac{2\gamma \sin\left(\frac{\left(\frac{5\pi }{6}-\alpha -m\right)}{m}\phi +\alpha \right)}{R\left(\sin\phi -D^{*}\right)} & \phi \in \left[m\right.,\left.0\right] \end{array}\right.$$

(5) $$\Delta P_{r}=\left\{\begin{array}{cc} \frac{2\gamma \sin\left(\frac{\left(\frac{5\pi }{6}-\alpha \right)}{m}\pi -\frac{\left(\frac{5\pi }{6}-\alpha +m\right)}{m}\phi +\alpha \right)}{R\left(\sin\phi -D^{*}\right)} & \phi \in \left[\pi \right.,\left.\pi -m\right]\\ \frac{2\gamma \sin\left(\frac{5\pi }{6}-\phi \right)}{R\left(\sin\phi -D^{*}\right)} & \phi \in \left(\pi -m\right.,\left.0\right] \end{array}\right.$$

*α* represents the water contact angle at *ϕ*  =  0 in positive direction, and the value of *α* is obtained in Figure 2c. The maximum values of Δ*P*_P_ and Δ*P*_r_ represent the critical breakthrough pressure in the positive (*P*_c − p_) and reverse (*P*_c − r_) direction, respectively. Through the analysis of Formulas (4) and (5), it is found that when *m* ≤ 1.215, the values of *P*_c − p_ and *P*_c − r_ are the same and equal to − 2*γ*/*D* (the critical breakthrough pressure of isotropic superhydrophobic membrane with the same pore size as Janus membrane). This means that when *m* ≤ 1.215, the obtained gradient Janus membrane does not have the anisotropic penetration resistance. When 1.925 > *m* > 1.215, *P*_c − p_ decrease with the increase of *m*, while *P*_c − r_ keeps the maximum value (− 2*γ*/*D*). When 1.925 ≤ *m*, *P*_c − p_ and *P*_c − r_ decrease with the increase of *m*, and *P*_c − p_ is always smaller than the *P*_c − r_. Combining the relationship between alkali treatment time and local texture angle *m* in Figure 2d, it can be found that the theoretical analysis results about the critical penetration pressure are consistent with the experimental results in Figure 3g. For convenience of expression, points A (solid‐liquid‐gas three‐phase contact point) and B (lowest point of liquid meniscus) are marked on the liquid meniscus (Figure 4b). For a conventional Janus membrane with gradient wettability within *ϕ* ∈ [*m*_1_,0] (Figure 4b), under a certain hydraulic pressure (*P*_H_), the three‐phase contact line (TCL) propels along the surface of the cylinder. When A reaches the position of *ϕ*  = *m*_1_ , the critical breakthrough pressure in positive direction is reached (*P*_c − p_ =  Δ*P*_P_(*m*_1_)). Once *P*_H_ exceed *P*_c − p_ =  Δ*P*_P_(*m*_1_), water penetration can be achieved. To ensure the spontaneous water penetration in the positive direction, that is, a small *P*_H_ (the pressure of single water droplet) can achieve penetration, *m* should be greater than 1.215 and close to *π* to achieve a small positive breakthrough resistance. However, a large value of *m* would result in a small critical pressure (*P*_c − r_) in the reverse direction. Thus, it seems impossible for a single‐layer Janus membrane with straight pore to have both positive spontaneous penetration performance and high *P*_c − r_. Here, the existence of *δθ* may provide a way to resolve this contradiction. When A reaches the position of *ϕ*  = *m*_1_ , point B has already entered the HL side due to the presence of *δθ*. But point B has no effect on *P*_c − p_ because it cannot touch the HL part. If we can eliminate *P*_c − p_ through the contact of point B with the hydrophilic part, the liquid penetration does not require *P*_H_ to exceed Δ*P*_P_(*m*_1_) in the positive direction. This means that the positive critical breakthrough pressure can be reduced without changing the hydrophilization depth. Based on this understanding, we rationally constructed a liquid‐assisted Janus membrane by prewetting the HL side of the conventional Janus membrane with water. With the advancing of the water meniscus in positive direction, water penetration can be achieved once B touches the prewetted water layer (Figure 4c). At this time A is still on the SHB area and has not reached the position where *ϕ*  = *m*_1_. The vertical distance between points A and B is *H*, and the *H* follows Equation (6)

(6) $$H\;=\frac{\left(D+R-R\sin\phi \right)\left(\tan\delta \theta -\sin\delta \theta \right)}{\sin\delta \theta \tan\delta \theta }$$

Assuming A at this time is at the position where *ϕ* is equal to *m*_2_, *P*_H_ only needs to exceed Δ*P*_P_(*m*_2_) and is not necessarily greater than Δ*P*_P_(*m*_1_) to achieve liquid penetration in positive direction. In the reverse HL to SHB direction, the auxiliary liquid does not affect the critical breakthrough pressure (Figure 4e). In summary, the auxiliary liquid can reduce the positive critical breakthrough pressure without affecting the critical breakthrough pressure in the reverse direction.

### 2.4 Potential Applications

**Figure 5:**
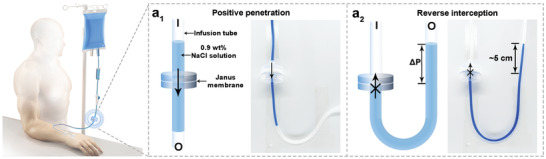
The liquid‐assisted Janus membrane is inserted into the infusion tube to prevent the blood backflow at the end of the intravenous transfusion. a_1_) During the infusion process, the liquid can quickly pass through the Janus membrane in the positive direction. a_2_) After liquid completely passed through the Janus membrane, the outlet side of the infusion tube was raised to simulate that the pressure in the blood vessel was greater than the pressure of the liquid in infusion tube. It was found that the liquid could not return through the Janus membrane in the reverse direction. I and O represent the inlet and outlet, respectively.

Intravenous infusion is a widely used medical technology, which can deliver medications and nutrition directly into a person's vein.^[^ ^15^ ^]^ However, at the end of the infusion, there may be blood backflow caused by the venous pressure in the body exceeding the liquid pressure in the infusion tube. If the treatment measures are not timely, the blood backflow will easily lead to the formation of blood clots and cause medical accidents. The Janus membrane with directional penetration “diode” performance is expected to solve the problem of blood backflow. Here, the Janus membrane with the SHB side facing the inlet is inserted into the infusion tube. The 0.9 wt% NaCl solution dyed with methylene blue is used for simulation experiments. Due to the positive spontaneous permeability and high flux, the liquid can quickly pass through the Janus membrane, which means that the presence of the Janus membrane will not affect the intravenous infusion process (**Figure** **5**a_1_). After liquid completely passed through the Janus membrane, the outlet side of the infusion tube was raised to simulate the venous pressure in the body exceeding the liquid pressure in the infusion tube. Due to the high critical penetration pressure in the reverse direction, the liquid cannot flow backward through the Janus membrane (Figure 5a_2_; Movie S5, Supporting Information), which means that blood backflow can be avoided. Furthermore, unidirectional penetration can also be used for controllable fluid transport (Figure S3, Supporting Information).

## 3 Conclusion

In summary, we introduce the liquid layer into the hydrophilic side of the single‐layer Janus membrane to form a liquid‐assisted Janus membrane. This solid‐liquid composite structure overcomes the contradiction between the positive and reverse critical penetration pressures of single‐layer Janus membrane with straight pore, that is, the spontaneous transport in the positive direction usually means weak critical resistance in the reverse direction. The auxiliary liquid can effectively reduce the critical breakthrough pressure in the positive direction without affecting the critical breakthrough pressure in the reverse direction, thereby achieving unidirectional water penetration with high pressure difference. We believe that this strategy can be appropriated for various single‐layer Janus membranes to enhance the unidirectional penetration performance. This water directional penetration “diode” performance with a high pressure difference have the potential application to prevent blood backflow during infusion, which further enhances the application value of Janus membrane.

## 4 Experimental Section

### Preparation of the Liquid‐Assisted Single‐Layer Janus Membrane

Copper mesh was firstly rinsed in ethanol and then soaked in 1 m HCl solution to dislodge surface oxides. After that, the cleaned copper mesh was placed into a mixture of 2.5 m NaOH and 0.15 m (NH_4_)_2_S_2_O_8_ at room temperature, forming blue Cu(OH)_2_ nanoneedle arrays on the surface. The Cu(OH)_2_ nanoneedle arrays coated mesh was immersed into the ethanol solution containing SH(CH_2_)_9_CH_3_ and SH(CH_2_)_10_COOH at different molar ratios for 12 h to obtain superhydrophobicity. The total thiol concentration was 1 × 10^−3^ m. To realize Janus wettability, the asymmetric decoration was conducted by simply floating the obtained superhydrophobic membrane on the NaOH solution surface. The stable air/water interface slows down the diffusion of NaOH solution, thereby forming a wettability gradient along the membrane. The hydrophilization depth can be adjusted by tuning the single‐face treatment time. Finally, the conventional Janus membrane with opposite wettability was obtained. On that basis, a liquid‐assisted Janus membrane is further constructed by prewetting the hydrophilic side of the Janus membrane with water.

### Characterization

The surface microstructures of the samples were observed by a scanning electron microscope (SEM, Quanta 250 FEG). An EDX detector was used to characterize the chemical composition. The contact angles were measured by an OCA20 machine (Data‐Physics, Germany) at room temperature.

## Conflict of Interest

The authors declare no conflict of interest.

## Supporting information

Supporting Information

Supplemental Movie 1

Supplemental Movie 2

Supplemental Movie 3

Supplemental Movie 4

Supplemental Movie 5

## Data Availability

Research data are not shared.
