# Supplementary material for: Liquid‐Assisted Single‐Layer Janus Membrane for Efficient Unidirectional Liquid Penetration
Source: Adv Sci (Weinh). 2021 Nov 10;9(2):2103765. doi: 10.1002/advs.202103765 (PMC8760174; doi:10.1002/advs.202103765)
Supplement: Supplementary file 1 — Supporting Information [file ADVS-9-2103765-s004.pdf]

WILEY-VCH

© 2021 Wiley-VCH GmbH

ADVANCED SCIENCE
Open Access

Supporting Information

for *Adv. Sci.*, DOI: 10.1002/advs.202103765

Liquid-Assisted Single-Layer Janus Membrane for Efficient Unidirectional Liquid Penetration

Zhihong Zhao, Yuzhen Ning, Shuang Ben, Xudong Zhang, Qiang Li, Cunming Yu, Xu Jin*, Kesong Liu* and Lei Jiang*

Supporting Information

Liquid-Assisted Single-Layer Janus Membrane for Efficient Unidirectional Liquid Penetration

Zhihong Zhao, Yuzhen Ning, Shuang Ben, Xudong Zhang, Qiang Li, Cunming Yu, Xu Jin*, Kesong Liu* and Lei Jiang*

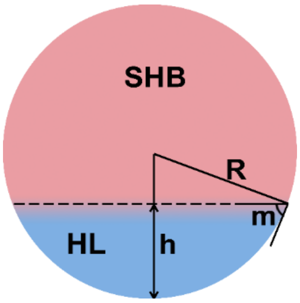

Figure S1. The relationship between the hydrophilization depth h and local texture angle m.

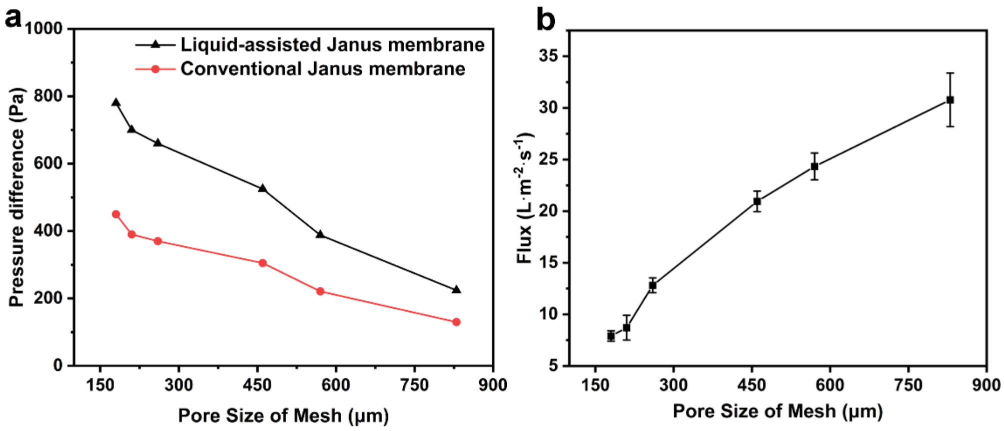

Figure S2. (a) When water can spontaneously penetrate in positive direction, pressure difference of the Janus membrane with different pore size. Compared with conventional Janus membranes, the liquid-assisted Janus membranes show water unidirectional penetration performance with higher pressure difference. (b) In positive direction, fluxes of Janus membrane with different pore sizes.

The pore size is the determinant of the critical breakthrough pressure of the porous membrane. The pressure difference of the conventional Janus membranes and the liquid-assisted Janus membrane increases with the decrease of the pore size. The pressure difference here refers to the pressure difference between the two sides of the membrane when water can spontaneously

penetrate the Janus membrane in positive direction. Because the auxiliary liquid can reduce the minimum thickness of the hydrophilic portion required for spontaneous water penetration in the positive direction, liquid-assisted Janus membranes exhibit higher pressure differences compared to conventional Janus membranes with different pore sizes (Figure S2a). This result shows that the auxiliary liquid has a lifting effect on the pressure difference of Janus membranes with different pore diameters. Although the reduction of the pore size can obtain a greater pressure difference, the reduction of the pore size will lead to the decrease of Janus membrane flux in positive direction (Figure S2b). In summary, in practical applications, we need to comprehensively consider pressure difference and flux of the Janus membrane to select the appropriate pore size.

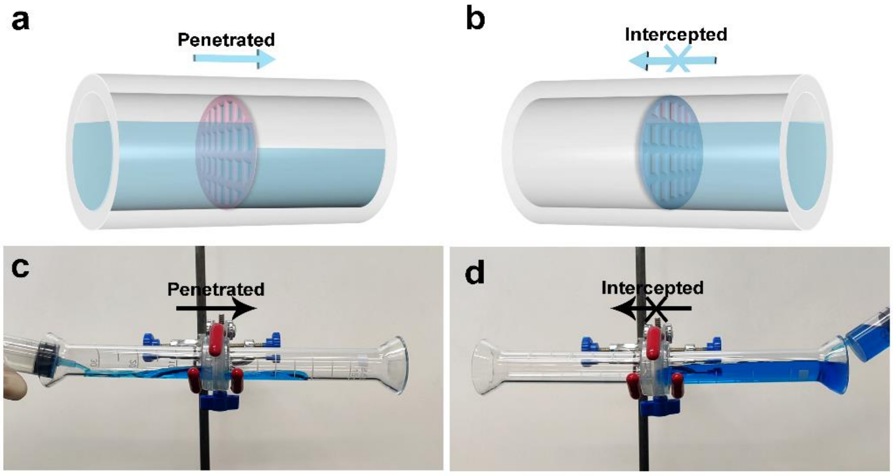

Figure S3. Unidirectional penetration is used for controllable fluid transport. (a−b) Schematic diagram of controllable fluid transport. (a) When water is injected from the SHB side, it can easily pass through the liquid-assisted Janus membrane. (b) When water is injected from the HL-W side, it cannot pass through Janus membrane due to the high reverse critical breakthrough pressure of the liquid-assisted Janus membrane. (c-d) Controllable water (dyed by methylene blue) flow direction. Here, the liquid-assisted Janus membrane obtained after 6 min of hydrophilization modification is used for the fluid transport test.

Movie S1. When the single-sided alkali treatment time is 6 min, water cannot spontaneously penetrate the conventional Janus membrane from SHB to HL-D direction.
Movie S2. When the single-sided alkali treatment time is 6 min, water spontaneously penetrates the liquid-assisted Janus membrane from SHB to HL-W direction.
Movie S3. When the single-sided alkali treatment time is 10 min, water spontaneously penetrates the conventional Janus membrane from SHB to HL-D direction.
Movie S4. When the single-sided alkali treatment time is 10 min, water spontaneously penetrates the liquid-assisted Janus membrane from SHB to HL-W direction.
Movie S5. Positive penetration and reverse backflow prevention. Due to the positive spontaneous permeability and high flux, the liquid can quickly pass through the Janus membrane. After liquid completely passed through the liquid-assisted Janus membrane, the outlet side of the infusion tube was raised to simulate the venous pressure in the body exceeding the liquid pressure in the infusion tube. Due to the high critical penetration pressure in the reverse direction, the liquid cannot flow backward through the Janus membrane.
